# Informing remediation of benzene contamination in drinking water distribution systems through multi-criteria decision analysis

**DOI:** 10.1016/j.hazadv.2021.100013

**Published:** 2021-11

**Authors:** Levi M. Haupert, Jon McDonnell, Kathy Martel, Michael D. Miles, Matthew L. Magnuson

**Affiliations:** a Office of Research and Development, Center for Environmental Solutions and Emergency Response, U.S. Environmental Protection Agency, 26 West Martin Luther King Drive, Cincinnati, OH 45268, United States; b The Cadmus Group, 100 Fifth Avenue Suite 100, Waltham, MA 02451, United States

**Keywords:** Analytic hierarchy process, Benzene, Decontamination, Drinking water, Multi-criteria decision analysis, Remediation

## Abstract

When contamination incidents occur in drinking water distribution systems, utilities need to select the remediation technologies most suited to their system-specific conditions and the contaminants of concern. Technology selection often involves balancing competing priorities. Multi-Criteria Decision Analysis (MCDA) is a promising approach that has been used extensively in other industries but not yet in drinking water system remediation. This paper discusses development of a computer-based tool that allows practitioners to leverage the Analytical Hierarchy Process (AHP), a well-established method of MCDA, to select remediation technologies based on their effectiveness and their compatibility with the practitioner’s project objectives. This paper focuses on benzene, a contaminant implicated for many years in contamination incidents following spills and, more recently, wildfires.

## Introduction

Contamination of drinking water distribution systems can interrupt service and pose a threat to human health. Contamination can happen as the result of ineffective treatment processes, poor repair practices, back-flow, malevolent acts, natural disasters, entry of animals through physical gaps (Angulo et al., 1997; Falco and Williams, 2009), or chemical spills into source water or soil adjacent to buried piping. For example, the 2014 chemical spill into the Elk River in Charleston, West Virginia affected drinking water for roughly 300,000 people (Rosen et al., 2014; Whelton et al., 2017).

Benzene is a prominent contaminant of concern for spills of gasoline, crude oil, and some other petroleum products. Related incidents affecting drinking water continue to occur. For instance, during a deadly 2021 winter storm in Texas, benzene contamination, perhaps resulting from backflow from an industrial source, left 101,000 residents without water (Douglas, 2021). When benzene is present in the soil adjacent to plastic pipes, as may occur with fuel spills, benzene can permeate from the soil through the pipe wall into the drinking water. The rate of permeation depends on the type of plastic, the pipe wall thickness, and other factors (Mao et al., 2010; USEPA, 2002; Whelton et al., 2010). Benzene contamination can also occur in water systems that have been damaged by wildfires (Proctor et al., 2020). The precise mechanism of contamination in these cases is still unknown. However, as described by Wilson (2019) in the case of wildfire damage in Paradise, California, the contamination can persist even after contaminated water has been flushed from the system.

Persistent infrastructure contamination poses several challenges for drinking water utilities. One of these challenges is selecting an appropriate technique/technology to remediate the system. Cost and efficacy are often deciding factors for selecting remediation strategies. However, these considerations are often subject to large uncertainties and arbitrary value judgements. There may be some disagreement for what efficacy threshold is acceptable—or indeed how exactly efficacy is defined. For instance, if one technique can reduce the contamination to well below a regulatory level, but a competing technique can reduce the contaminant level below the analytical reporting limit, how much should that difference affect the decision? Furthermore, considerations about less-tangible metrics such as stakeholder perception often affect the decision-making process. These aspects of decision-making can often be non-obvious and become protracted when *ad hoc* decision processes are used. For example, the City of Santa Rosa, California, USA documented their 2018 response to wildfire-induced benzene contamination affecting 13 homes in their Fountaingrove neighborhood (City of Santa Rosa, 2019). To completely restore water quality, their process, which appears *ad hoc* (created for the particular purpose), took about 8 months, which is not surprising because specialized tools for water system remediation were not available.

Several decision process systems for multi-criteria decision analysis (MCDA) can provide organization and stepwise accountability for judgements regarding important environmental decisions. General reviews of decision process systems and their application to environmental decision making can be found in Toth and Vacik (2018); Huysegoms and Cappuyns (2017); Huang et al. (2011); and in Kiker et al. (2009). These reviews compare and contrast various MCDA systems, of which there are dozens, many of which rely on mathematical approaches of varying complexity. One of the most popular MCDA systems is the analytical hierarchy process (AHP), developed by Saaty (1988), (2008). AHP uses pairwise comparison matrices to compute sets of linear weights which are used to rank options by desirability (or priority). AHP has been used to make decisions on remediation options for contaminated sites (Critto et al., 2006; Li et al., 2018), but these applications have mainly focused on contaminated soil or bodies of water, not drinking water distribution systems. To date, there are no published MCDA tools or applications that support decision-making for remediation of drinking water infrastructure. A feature of water infrastructure decontamination, as compared to contaminated sites, is that water infrastructure is critical to the community it serves, so an applicable MCDA approach should be designed for rapid implementation and be adaptable to the evolving nature of water contamination incident response.

The purpose of this paper is to present an AHP-based tool developed to assist decisionmakers in prioritizing appropriate remediation technologies for benzene-contaminated drinking water infrastructure. The tool described herein is freely available in the Supplemental Material and implemented in Microsoft Excel. The tool is intended to increase consistency and transparency in the decision-making process.

## Methods

### Tool development

#### Software implementation

The AHP-based tool, referred herein as Water-APT (for Analytical Process Tool), was implemented in Excel (Microsoft Office 365 version), chosen because it is easy-to-use and widely available. Excel contains functions and features that perform the linear (matrix) algebra operations that underlie AHP calculations. Water-APT only uses functions from the standard Excel library. Water-Apt does not use macros, nor does it depend on any Add-ins. The discussion below includes some examples of this matrix algebra for the benefit of readers interested in the mathematical logic behind AHP (Saaty, 2008). Water-APT performs the necessary calculations automatically, meaning that an understanding of linear algebra is not required to use Water-APT. Via Excel, Water-APT can easily be used to evaluate contamination incidences at multiple sites across a distribution system by saving multiple versions of each run. This is important because many sites within a distribution system can be contaminated, and each site may require individual evaluation. The Excel file containing Water-APT is included in the supplemental material.

#### Subject matter expert panel

Water-APT was developed with feedback from an expert panel of water system practitioners to enhance its usability and applicability. Subject matter experts were selected based on experience with drinking water or wastewater systems, infrastructure decontamination, and operation. Their experience included response to incidents leading to benzene contamination, including spills of petroleum products and wildfires. Through a series of meetings, experts were asked to address the format, content, sophistication, and real-world applicability of Water-APT. They provided comments on terminology, user instructions, tool functionality, formatting, and overall tool organization.

### Logic flow of multi-criteria decision analysis methodology

**Fig. 1.**
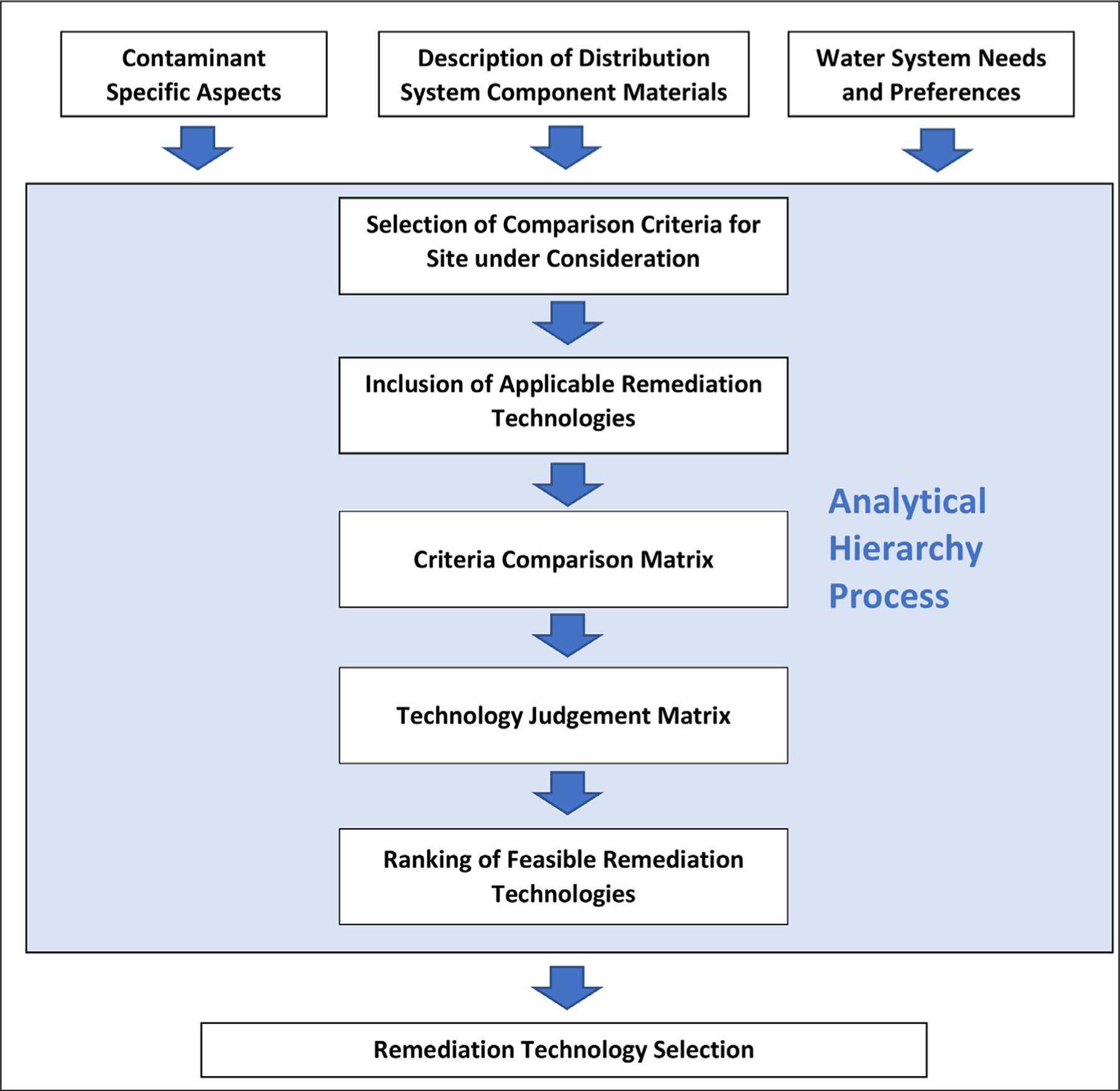
Logic Flow of Water-APT. Information inputs into the decision process are shown at the top of the figure. Information from the inputs flows into the shaded area which encloses the steps in the AHP implementation of Water-APT. The output of the AHP process then feeds into the decision objective, which is selection of the remediation technology.

Water-APT guides the user through several steps (Fig. 1) to complete the MCDA methodology and achieve the goal of ranking of remediation technologies for each evaluated contamination site. It first requires users to input system-specific information, namely the materials that compose the site within the distribution system that is being evaluated. Although Water-APT can be applied to any system component, it specifically calls out pipe materials because these generally constitute the bulk of a water system construction materials by mass. Consideration of other components by Water-APT is discussed below in the “Determination of Applicable Remediation Technologies” section. Water-APT then presents useful information for the user’s consideration as they move through each subsequent step. Each of the steps visualized in Fig. 1 is described below in a separate subsection.

Similar to the approach taken by Critto et al. (2006), users are asked to select the criteria against which each remediation technology will be evaluated. Next, users are presented with a series of remediation technologies that are stored in Water-APT’s underlying database. All remediation options are displayed and Water-APT indicates which may be feasible (i.e., has some chance of success) based on user-entered systemspecific characteristics. Should the programming in Water-APT mischaracterize the feasibility of a particular option, users can override this determination. Once feasible remediation technologies are selected, users are asked to indicate the relative importance of each selected criterion using a pairwise comparison matrix. Finally, the user judges how each technology might perform against each selected criterion for their system.

#### Selection of comparison criteria for site under consideration

The AHP process is based on systematically comparing criteria that are important for the decision being made. Thus, selection of criteria is the first step in Water-APT (Fig. 1), and the user selects criteria based on their knowledge of the water system under consideration. Water system remediation is infrequent, so it is not an activity many users will be familiar with. An important part of Water-APT is that it assists users who may not be familiar with criteria important during remediation. Water-APT does this by including 14 criteria, summarized in Table 1, that the expert panel of water system practitioners (described above) identified as being the most relevant for water utilities faced with remediation challenges. The criteria may reflect possible regulatory requirements (drinking water quality, waste management, environmental impact), internal goals (e.g., energy efficiency, water efficiency, cost), safety concerns (operators, contractors, public), and/or customer concerns (e.g., aesthetics, technology acceptance). Table 1 provides general descriptions and considerations regarding each criterion to guide the user; these considerations should not be considered prescriptive or exhaustive. Thus, users are also given the option to write-in two customized criteria if the provided criteria do not adequately represent the water system priorities and water customer concerns.

To begin using Water-APT, the user selects from Table 1 between 5 and 9 criteria for each analysis as recommended by Saaty and Ozdemir (2003). Selecting more criteria will increase the accuracy of the analysis; however, using too many criteria could lead to user fatigue. Water-APT triggers an error message window and prompts users to refine their selections if the number of criteria selected is less than 5 or greater than 9.

#### Inclusion of applicable remediation technologies

In this step, the user instructs Water-APT to include in the AHP analysis technologies that are potentially applicable to the site, which are referred to as “feasible” technologies in Water-APT. “Technology” in this case can refer to a device or method applied for remediation of the water system itself, not for the source of the benzene. For example, nearby benzene in soil can permeate and contaminate water pipes (USEPA, 2002); sources of benzene may require separate remedial techniques. Because there is little literature on the topic of distribution system remediation for most potential contaminants, including benzene, Water-APT assists the user in several ways.

First, Water-APT contains a comprehensive database of remediation technologies that may be feasible for benzene. Table 2 summarizes the major types of technologies that have been utilized for water system remediation for benzene, as well as a range of other contaminants, based on a literature search (Szabo and Minamyer, 2014) and full-scale experiments (USEPA. 2021) conducted by EPA. The first five technologies listed in Table 2 have been utilized for remediation of benzene, either during actual incidents or in controlled studies.

Second, Water-APT’s programming and logic reflects the property of benzene that contributes heavily to its persistence and corresponding need for decontamination, namely the ability to permeate plastic pipe. Glassy polymers, such as rigid PVC, are resistant to permeation except at high concentrations (Berens, 1985). However, other polymers are assumed permeable. Once plastic pipes are permeated, it is possible that desorption from the pipe wall (rather than transport due to rapid, continuous flushing) determines the rate of decontamination by flushing (Haupert and Magnuson, 2019). Therefore, if the Water-APT user indicates that the system contains (for example) polyethylene pipes, Water-APT automatically includes intermittent flushing as a feasible remediation technology.

Third, while Water-APT automatically includes the first five technologies as feasible, it provides the user with capability to include other decontamination technologies reported to be effective for other contaminants, and potentially applicable for benzene. Other technologies may contribute to achieving other goals besides remediation of benzene, so it is important to alert the user to technologies that may optimize the outcome of the remedial process. For instance, some technologies (Table 2) may also achieve rehabilitation of aged infrastructure, a pressing issue faced by many utilities. Thus, a user may be interested in including a technology that helps achieve both the short-term goal of remediating benzene and the long-term goal of infrastructure improvement.

Finally, Water-APT allows users to input a technology of their choosing. For example, novel technologies might be proposed by researchers or vendors, e.g., for a particularly challenging site. Often, particularly after contamination incidents which achieve widespread attention, decision makers are overwhelmed by researchers or vendors interested in assisting the water utility. Including the novel technology enables the AHP process to provide a systematic and transparent comparison of the novel technology with other options.

#### Pairwise criteria comparison matrix

##### Populating the pairwise matrix.

Next, the user completes a pairwise criteria matrix, which is fundamental to the AHP process. The Water-APT software forms a blank matrix based on the user selected criteria (Section 2.2.1) by presenting them in both the rows and columns, as shown in Table 3. The user populates the initially blank matrix by comparing the relative importance of the criteria based on the user’s preference and the remediation objectives. To do this, the user works across each row in Table 3, asking themselves the question “How much more important is criteria in the row compared to criteria in the column, e.g., “How much more important is it that a particular remediation approach be water efficient than for it to have long-term effectiveness?” Because AHP uses mathematics, the answer to this question needs to be expressed as a number. Table 4 provides a fundamental scale that converts the user’s knowledge and experience to a numerical value. The benefits and scientific rationale of using this particular numerical scale have been described in detail by Saaty (1980, 2000). Therefore, if the user answers, “I think that water efficiency will be moderately more important that long term effectiveness,” then the user inputs a “3” in the respective cell of the matrix.

In the Water-APT software, the user populates the upper right half (above the stair-step line) using drop down menus, and the lower left half is automatically calculated using the principles of linear algebra in that the comparison matrix (*A*) is a reciprocal matrix, so $A_{i,j}=1/A_{j,i}$ where $A_{i,j}$ is the pairwise comparison value where the i*th* row meets the j*th* column.

Once the pairwise comparison matrix is complete, Water-APT calculates a set of weights (*w*) for each criterion. These weights are later used in combination with the technology judgement matrix (described below) to form the final rankings of the technologies described below. A simple normalized column sum technique, which is comparatively easy to implement and offers consistently good performance (Choo and Wedley, 2004), was selected to calculate the criteria weights. A useful first step for this method is to first compute an intermediate matrix *B*, which is constructed by independently normalizing each column of *A*:

(1) $$B_{i,j}=\frac{A_{i,j}}{\sum_{i}A_{i,j}}$$

If there are n criteria, the weight for the *i*th criterion is then given by:

(2) $$w_{i}=\frac{1}{n}\sum_{j=1}^{n}B_{i,j}$$

##### Water-APT features to help improve usefulness of weights and ease of use.

The weights are at the heart of the AHP, but the usefulness of the generated weights depends on the quality of the pairwise comparison matrix used to derive them. Users can make errors when filling out the matrix. Errors can range from simply inputting an unintended value to making contradictory selections between two sets of pairwise comparisons due to the complexities of balancing competing concerns. These errors lead to inconsistency in the pairwise comparison matrix. Notably, it becomes increasingly difficult to make consistent comparisons between criteria as the number of criteria increases (Saaty and Ozdemir, 2003). Users are advised to think carefully about selecting only criteria that are relevant to their analysis, so as not to unnecessarily complicate the analysis.

To measure the consistency achieved in the pairwise comparison matrix, Water-APT calculates a consistency ratio (*CR*) value and displays a warning to the user when the CR values gets too large. It is commonly accepted that a *CR* value less than 0.1 signifies reasonably consistent pairwise comparison values (Saaty and Ozdemir, 2003). The first step in this process is for Water-APT to calculate the consistency index (*CI*) using the number of criteria (*n*) and the maximum eigenvalue of the pairwise comparison matrix ($\lambda_{max}$) according to the formulas:

(3) $$CI=\frac{\lambda_{max}-n}{n-1}$$

(4) $$\lambda_{max}=\frac{1}{n}\sum_{i=1}^{n}\sum_{j=1}^{n}A_{i,j}\frac{w_{j}}{w_{i}}$$

For a consistent criteria comparison matrix, $A_{i,j}w_{j}/w_{i}=1$ and $\lambda_{max}=n$.

Next, an average random consistency index is used to determine the appropriate random consistency indicator (*RI*) based on the corresponding value for n (Table 5). The RI values in Table 5 are taken from Saaty (1980, 2000). They are widely used in current practice, often with little modification (Franek and Kresta, 2014).

Water-APT then calculates the *CR* value:

(5) $$CR=\frac{CI}{RI}$$

To help alleviate the difficulty of reaching consistency in the pairwise comparison matrix, a novel aspect of Water-APT, compared to many AHP tools, is that it presents users with the closest consistent matrix using a linearization technique described by Benítez et al. (2011,^2014^). Users may opt to use the automated consistent criteria matrix by indicating their preference from a drop-down menu after completing the pairwise comparison matrix. The closest consistent matrix *X* to the initial guess matrix *A* is given by:

(6) $$\log\left(X\right)=\frac{1}{2n}\sum_{i=1}^{n-1}\frac{\text{trace}\left(\log{\left(A\right)}^{T}\phi \left(y_{i}\right)\right)}{y_{i}{}^{2}}\phi \left(y_{i}\right)$$

The $y_{i}$ in Eq. (3) is the *i*-th column vector taken from the matrix $Y_{n}$, which is defined by following the pattern:

(7) $$Y_{2}=\left(\begin{array}{c} 1\\ -1 \end{array}\right),Y_{3}=\left(\begin{array}{cc} 1 & 1\\ -1 & 1\\ 0 & -2 \end{array}\right),Y_{4}=\left(\begin{array}{ccc} 1 & 1 & 1\\ -1 & 1 & 1\\ 0 & -2 & 1\\ 0 & 0 & -3 \end{array}\right),\ldots$$

The function ϕ generates a matrix from a column vector according to the formula:

(8) $${\left[\phi \left(y\right)\right]}_{j,k}=y_{j}-y_{k}$$

#### Technology judgement matrix evaluation

Before the final set of criteria weights can be used to assign priorities to the technologies, the performance of technology options should be evaluated with respect to the criteria. In Water-APT, this step is accomplished using a single technology judgement matrix that ranks how each technology performs against each selected criterion (Chattopadhyay and Ramanathan, 1998; Ramanathan, 2001; Saaty, 1987). Table 6 provides an example of a completed judgement matrix corresponding to the criteria in Table 3. The elements of the technology judgment matrix elements $\left(S_{i,j}\right)$ score the *i*th option with respect to the *j*th criterion.

The user is asked to score each technology based on its performance with respect to each criterion using an absolute numerical scale from 1 to 5. Higher numbers correlate with better performance. To do this, this user is instructed to consistently utilize criteria-specific definitions used for technology judgement matrix scoring (which are found in Table S1). Depending on the criterion, the scoring language can range from objective to subjective, due to unknown or unpredictable factors related to applying the technology at a particular site. However, part of the power of the overall AHP approach, leading to its acceptance in many applications, is its ability to help lead to reasonable results (in this case, technology selections) in the absence of complete data.

#### Prioritize technologies

Finally, Water-APT calculates the priority ( *P* ) for the *i*th technology option as:

(9) $$P_{i}=\sum_{j=1}^{n}S_{i,j}w_{j}$$

Water-APT presents users with ranked remediation options and uses a visual cue to indicate which was determined to be the most advantageous based on the AHP analysis. The priorities calculated from the example matrices given in Tables 3 and 6 are discussed below.

## Results

It is useful to consider a hypothetical case study, including several scenario variations in which the user changes input variable to adapt to the evolving incident, to illustrate the versatility of Water-APT to help prioritize remediation technology. The premise of this case study is inspired by real events, but the evaluation and selection of technologies is fictional, for illustrative purposes only. Also, the parties that will need to make decisions about remediation technologies and will therefore find Water-APT useful will vary from case-to-case and site-to-site. These parties could include not only local governmental and water industry personnel, as described in the case study below, but also consultants, state or federal officials, and others.

### Scenario base-case: Description and system-specific characteristics

During a hypothetical intense wildfire in northern California, several homes and businesses were severely burned after residents were safely evacuated. Once residents returned to their homes, the local water utility began receiving complaints about odorous water coming from the tap. Sampling was conducted and showed benzene levels in exceedance of California’s Maximum Contaminant Level (MCL) throughout the area. Benzene had not been detected before the wildfires. In response, the water system’s remediation planning technical group (RPTG), in collaboration with state and local officials determined that remediation options needed to be evaluated promptly and deployed the Water-APT to help aid their decision-making process. The RPTG will likely consist of several members because it is uncommon for a single expert to have comprehensive knowledge of all the technical issues involved. In practice, the composition of the RPTG will vary with the specifics of the incident and may change as the remediation process evolves. In addition to the water utility itself, which will be very familiar with the system, the RPTG could also involve regulators, consultants, and/or other experts in technical challenges of remediation at particular sites. The AHP process that Water-APT utilizes has the benefit of being able to aggregate the contributions of multiple experts during the process of completing the pairwise comparisons and the technology judgement matrix (Ossadnik et al., 2016).

The RPTG began by entering system-specific characteristics into Water-APT. In this incident, the contaminant of concern was benzene, and the pipe materials at the contamination site were determined to be primarily polyvinyl chloride (PVC), polyethylene, and ductile iron. Following the input of system-specific characteristics, the RPTG selected the criteria they thought most important to consider when comparing remediation options. The RPTG made the following selections, entering the accompanying justifications into the notes section of Water-APT (alternate selections, and their effects, are discussed in scenario variants described in sections 3.2, 3.3, and 3.4):

- Criterion 1:
  ○ Water Efficiency
  ○ **Justification:** The community was experiencing a severe drought and local water resources were limited.
- Criterion 2:
  ○ Long Term Effectiveness
  ○ **Justification:** The region is subject to wildfires, so the county wants to make sure that the system will not be contaminated again at the time of the next fire. The water utility has learned that the way benzene contaminates the system is unclear, and that it could have resulted from contaminated air being sucked into the system when it lost pressure, or possibly as a result of melting of plastic pipes, or a combination of causes.
- Criterion 3:
  ○ Public Safety
  ○ **Justification:** Residents in the area had recently been affected by severe wildfires and county officials wanted to reduce any possibility of further physical harm to residents.
- Criterion 4:
  ○ Aesthetic Issues with Finished Water
  ○ **Justification:** Residents complained about odorous tap water when the contamination incident occurred. To rebuild public trust in the safety of water supplies and ensure the success of the remediation effort, aesthetic issues of finished water should be limited to the extent possible.
- Criterion 5:
  ○ Customer Acceptance
  ○ **Justification:** Customers have raised complaints about other routine system maintenance when it affects water service pressure or water supply, and the utility wishes to minimize the inconvenience and disruption of service that may accompany system remediation.

The RPTG considered all remediation options suggested by default by Water-APT (i.e., the first five in Table 2) to be technically feasible and so did not override Water-APT’s feasibility determination, prompting Water-APT to include these remediation options in the subsequent AHP analysis worksheet. Using Water-APT on a mobile device, the RPTG, in consultation with county officials, made inputs into the 5-by-5 pairwise criteria matrix (the first five rows and columns of Table 3) generated by Water-APT for the five criteria above. The RPTG next assigned scores (the first five columns of Table 6) to each remediation option based on the option’s performance against the five criteria.

The first column of Table 7 shows the results of the AHP analysis for the "initial case", meaning it was based on the original assumptions and inputs of the RTPG. Additional columns of Table 7 show the results of the scenario variants discussed below. In these variants, the RPTG changes inputs in the AHP analysis based on the evolving nature of the incident, in which additional information has become available. Each remediation option is given a priority ranking (Eq. (9)) based on the RPTG’s inputs. The RPTG used the relative rankings to discuss the remediation possibilities with the county officials. During the discussion, one of the officials revealed that, contrary to prior beliefs, the cost of the remediation would not be paid by the state unless there was a compelling justification to do so. The wildfires that year were numerous, and there was great demand on limited finances.

### Scenario variant: With cost

With this new information, the RPTG, within minutes, included costs in the criteria comparison and technology judgement matrices (Tables 3 and 6), and saved the file under a new, descriptive name for ease of comparison with the base-case. With a larger number of criteria factors, it becomes more difficult to mentally balance all the factors. The RPTG noticed Water-APT generated a warning about matrix consistency (*CR* = 0.33), so the RPTG activated the automatic consistency feature. The RPTG compared results with and without the automatic consistency feature, and the relative order was reassuringly similar.

The RPTG shared the results (Table 7, “With Cost” column) with the county officials, were inclined to pursue intermittent flushing as a general approach across the county. However, they noted two cases needing further analysis. First, there was concern that flushing the system may not be accepted by the public as being effective enough for the local elementary school. Second, it was also noted that some parts of the affected area were not served by public sewers, and run-off in those spots would affect sensitive downstream areas.

### Scenario variant: With cost, for a school

In this variant, the school was essentially considered a separate location, with its own pairwise comparison matrix and technology judgement matrix. The RPTG entered a set of pairwise criteria (the first six rows and columns of Table 3), and then increased the importance of customer acceptance for each relevant entry (Table 8). These inputs reflected that, a few years ago, the school experienced some concerns about lead, so a POE system was installed, even though POU/POE systems were not common in the community due to maintenance requirements. Likewise, the criteria comparison matrix was changed to reflect less concern about cost and more concern about customer acceptance. Changes to the judgement matrix (new values in Table 9) reflected that customers have already accepted the POE system, and also that the school was a much smaller system, so costs were less of a concern than in the city-wide case. In addition, the costs for POE would be minimal, since a system is already installed at the school, and it would need adapted for benzene. The RPTG determines that this would be efficacious based on availability of cartridges certified for benzene and lead removal with sufficient capacity based on levels of benzene and lead measured in the influent water. As an additional precaution, the RPTG suggests the samples of the POE treated water be sent periodically for laboratory analysis to verify the certified performance.

Water-APT provided a transparent indication that POU would be the leading contending technology at these sites (“With Cost, for a School” column of Table 7). In preparing a justification to the state, the RPTG cited the Water-APT recommendation, adding that it could be implemented very quickly because a POE was already installed, although it required the selection of suitable media.

### Scenario variant: With cost and waste management

For the second concern raised by the county officials, the RPTG included waste management in the pairwise comparison matrix (Table 3) and the judgment matrix (Table 6). The results revealed a virtual tie between pipe replacement and intermittent flushing in the areas not served by public sewers.

Waste management can be a complex issue under the best of circumstances, and it becomes increasingly challenging when hazardous materials may be present. Often, waste issues intertwine with other criteria. As it happened, the matrix the RPTG constructed had poor consistency. Ties like the one encountered by the RPTG here are one potential failing of inconsistent criteria comparison matrices, which often contain cyclic preference ordering. Using the auto-consistency feature of Water-APT eliminated the inconsistency and also eliminated the tie. The new consistent matrix gave results (final column of Table 7) that showed a clear recommendation of pipe replacement in the areas without sewers. These Water-APT results were documented in the justification prepared for the state.

## Conclusion

This paper introduces and demonstrates Water-APT as a tool for guiding decision makers toward the selection of optimal technologies to remediate water systems contaminated with benzene. Water-APT is based on AHP, which as discussed in the introduction, has inherent benefits that have contributed to its continued popularity as a tool for decision-making in many fields. These benefits include ease of use, along with several features that make the selection process structured, transparent, scientific, and explainable.

Water-APT also contains available benzene remedial technology information and enables flexibility to add customized remedial technologies. Its ease of use is important because many sites within a distribution system can be contaminated, and each site may require individual evaluation. Very importantly, Water-APT helps the user rapidly adapt remedial technology selection to new information that becomes available as the contamination incident evolves.

The ease of use means that the tool can be used rapidly and iteratively in the initial stages of an incident when time is critical and concerns are evolving. The AHP methodology helps the Water-APT user balance the expectations of the communities served, competing and complex criteria presented by the water system design and operation, the lack of data inherent for unexpected contamination incidents, uncertainties in such data, and the changing data needs of the evolving incident.

These benefits may streamline responses to future benzene contamination incidents because Water-APT incorporates real life lessons learned from prior incidents, in part drawing on the experiences of the subject matter expert panel who contributed to the development of the tool. To this end, as mentioned previously, the City of Santa Rosa, California, USA experienced wildfire-induced benzene contamination affecting 13 homes (City of Santa Rosa, 2019). To completely restore water quality, their process, which appears *ad hoc* (created for the particular purpose), took about 8 months, which is not surprising because specialized tools for water system remediation were not available. Other systems impacted by subsequent wildfires benefited from Santa Rosa’s experience (PID, 2020), and response to future incidents may benefit from tools such as Water-APT that enable transfer of experience.

The tool could be also be straightforwardly adapted for use with contaminants other than benzene, although it may be desirable to have data to best fill out the technology judgement matrix for those other contaminants. The effectiveness for the Water-APT as a decision-making tool for benzene is enhanced by the availability of studies of past cases of benzene contamination. The tool could, accordingly, work equally well for other well-studied contaminants. Because the AHP process in Water-APT mathematically helps deal with uncertainties, the tool could also, however, be usable for contaminants with less technical data. Indeed, the ultimate priority of a technology is a balance of technical and non-technical criteria, so improving the quantity of technical data may not impact the ultimate decision.

## Supplementary Material

Supplementary Material 1

Supplementary Material 2

## Figures and Tables

**Table 1:** Criteria used as the basis for the Water-APT tool. Relevancy questions are provided to help users select the most appropriate criteria.

Criterion	Relevancy questions
Aesthetic Issues with Finished Water	Will the taste, odor, or appearance of the finished water change as a result of remediation? For example, will its taste and smell change beyond seasonal norms?
Customer Acceptance	How important is public and customer acceptance of a remedial approach? Will customers accept the need to interrupt service temporarily to perform this remediation? How do customers perceive risks associated with the remediation relative to risks of the contaminant?
Energy Efficiency	Are there energy efficiency goals or limitations that may be affected by the remediation operation?
Environmental Impact	Does remediation negatively affect environment? Will the remediation process result in a wastewater or chlorinated water discharge that affects the soil, plants, or animals?
Human Health Impact	Is there a risk that customers could ingest or inhale water containing residuals from the remediation process, such as corrosion products, additives, disinfectants, disinfection byproducts?
Impact on Infrastructure	How important are unintended consequences of remediation (e.g., corrosion) to the piping or other system components?
Life Cycle Cost	What are the capital, labor, and materials costs for remediation? How does the remediation process affect operations and maintenance costs?
Long-term Effectiveness	Will remediation permanently remove or reduce the contaminant level below desired limits or will additional corrective actions be needed in the future?
Operator or Worker Safety	Does remediation pose safety risks (e.g., exposure to chemicals or physical hazards) for the system operators or remediation workers? Do the operators and workers have adequate training, written procedures, and personal protective equipment to ensure a safe working environment?
Public Safety	Will the public be protected from any potential safety hazards (e.g., chemical exposure, high velocity water flushing) during the remediation work?
Regulatory Impacts and Considerations	Is the water system classified as a public water system? If not, will it be classified as a public water system in the future if supplemental treatment is added as part of the remediation? Will implementing remediation negatively affect compliance with Safe Drinking Water Act and/or other regulations?
Timeframe	Is the timeframe required for implementing remediation acceptable? Will the time required for related customer notifications, valve operations, equipment setup and removal, and/or water quality monitoring negatively impact current operations?
Waste Management Considerations	If wastewater (e.g., flushing water) is generated from this remediation process, will the disposal method meet all applicable environmental regulations? Will there be any solid wastes, like spent filters or point-of-use (POU) treatment elements? Will any wastewater or remediation wastes (e.g., replaced pipes) need treatment prior to disposal? If so, can this be done on site or will it require hauling to a specialized facility?
Water Efficiency	Are there water efficiency goals or limitations that may be affected by the remediation operation?

**Table 2:** Remediation technologies applicable to water distribution systems contaminated with benzene.

Remediation Technology	Applicability to Benzene Contamination
Component Replacement	This option involves determining the extent of contamination, isolating the affected part of the distribution system, replacing the component, and returning to service following established procedures. If the component is vulnerable to permeation by the contaminant, e.g., for benzene with some plastic/polymer components, any contaminated soil should also be removed before the new component is installed to avoid potential recontamination.
Continuous Flushing	Continuous flushing involves running large volumes of water for extended periods of time (e.g., hours or days). If the contaminant is dissolved in the water and does not adhere to or absorb into the water system pipe/component, the contaminant is carried out of the system along with the contaminated water. If the contaminant does adhere to or absorb into the component, e.g., for benzene with some plastic/polymer components, it may be necessary to extend the flushing duration.
Intermittent Flushing	Intermittent flushing can achieve simultaneous goals of remediation of contaminants and reducing water usage (Haupert and Magnuson, 2019) compared to continuously running water. Intermittent flushing is accomplished by isolating the pipes containing contaminated water and stagnating (i.e., holding) the water for a fixed period of time (e.g., hours) to allow the contaminant to desorb from the pipe wall into the bulk water. The contaminated water is then removed from the pipes by replacing the water (e.g., running it for minutes). For benzene, some systems have used a 72-hour stagnation period (Proctor et al., 2020). However, the site-specific stagnation period should consider system-specific factors like pipe materials, water-usage patterns, and cost/availability of labor and water.
Flushing with an Additive	Some additives can reduce the adherence of contaminants to wetted surfaces, which can enhance continuous or intermittent flushing. Casteloes et al. (2017) found that flushing with a pure water Alconox^®^ detergent solution removed all benzene, toluene, ethylbenzene, and total xylenes (BTEX) from copper pipe, and the detergent caused minimal changes to the physical and mechanical properties of ethylene propylene diene monomer, high density polyethylene, low density polyethylene, type-a cross linked polyethylene, rigid polyvinyl chloride, and chlorinated polyvinyl chloride. It should be considered that the additive itself may lead to a need for further flushing, as was observed for one surfactant evaluated for benzene remediation (USEPA, 2016).
POU/POE devices	A point-of-use (POU) device is a treatment unit (e.g., filter cartridge) installed at a specific faucet, showerhead, or other point of water use. A point-of-entry (POE) device is a treatment unit installed at the entry point to a water distribution system or on a customer service line (i.e., a whole house system). Devices approved for benzene removal by certification organizations are available (USEPA, 2006).
Unidirectional Flushing	Unidirectional flushing (also known as “scouring”) is similar to continuous flushing, except that it is accomplished by operating valves in sequence to produce high flushing velocities that can scour the pipe surfaces and lift sediment into the bulk water. Unidirectional flushing may be particularly effective for contaminants that are sorbed to this sediment or other solids. Benzene may sorb when such sediments/solids have organic content.
Shock Hyperchlorination	Shock hyperchlorination is the injection of chlorine to achieve a level of 20–50 mg/L of free chlorine (as chlorine). After a sufficient contact time, the water is flushed. This technique may be applicable to benzene contamination if involvement of biofilm or other substances amenable to oxidation are suspected.
Heated Flushing	The heated flush remediation option is used in building water systems to control *Legionella* bacteria (USEPA, 2016), and it may be applicable to benzene contamination to increase the kinetic rate of benzene desorption. Heated flushing may also overcome smaller desorption rates resulting from low seasonal water temperatures
Pigging	Pigging may be useful if benzene is suspected of being entrained on pipe surfaces, e.g., solids potentially introduced during wildfires. Conventional pigging (i.e., hard body pigging) is a technique used to mechanically scrape biofilm, sediment, soft scale, and loose deposits from the inside of a pipe with a mechanical cleaning device (USEPA, 2019).
Relining	Relining is often used in conjunction with pigging for pipe rehabilitation. Several relining techniques are available depending on the pipe (USEPA, 2019).
User-Defined, Novel Technology	This option represents novel or unconventional approaches to benzene decontamination.

**Table 3:** Example of Pairwise Comparison Matrix for a Seven-Criteria AHP Analysis. Each entry in the table describes the relative importance of a row criterion over a column criterion. The linguistic meaning of the entries is described in Table 4. The Water-APT user populates only the upper right; the lower left is calculated automatically.

Criterion	Water Efficiency	Long term Effec-tiveness	Public Safety	Life Cycle Cost	Aesthetic Issues	Customer Accep-tance	Waste Manage-ment
**Water Efficiency**	1	3	9	5	1/3	1	9
**Long-term Effectiveness**	1/3	1	3	1	1/3	1/5	1
**Public Safety**	1/9	1/3	1	1/5	1/7	1/9	9
**Life Cycle Cost**	1/5	1	5	1	5	3	3
**Aesthetic Issues**	3	3	7	1/5	1	3	5
**Customer Acceptance**	1	5	9	1/3	1/3	1	1
**Waste Management**	1/9	1	1/9	1/3	1/5	1	1

**Table 4:** Pairwise Comparison Values and Their Linguistic Meaning (Saaty, 1980, 2000).

Pairwise comparison values*	Linguistic meaning
1/9	The column criterion is extremely more important than the ***row*** criterion
1/7	The column criterion is very strongly more important than the ***row*** criterion
1/5	The column criterion is strongly more important than the ***row*** criterion
1/3	The column criterion is moderately more important than the ***row*** criterion
1	The ***row*** criterion is equally as important as the ***column*** criterion
3	The ***row*** criterion is moderately more important than the column criterion
5	The ***row*** criterion is strongly more important than the column criterion
7	The ***row*** criterion is very strongly more important than the column criterion
9	The ***row*** criterion is extremely more important than the column criterion

* Any number between 1/9 and 9 may be used to accommodate subtleties between the linguistic meanings. However, the listed values are often viewed as more useful for AHP.

**Table 5:** Average random consistency index RI (Saaty, 1980, 2000)

*n*	1	2	3	4	5	6	7	8	9	10
***RI***	0.00	0.00	0.58	0.90	1.12	1.24	1.32	1.41	1.45	1.49

**Table 6:** Example of technology judgement matrix with seven criteria and five feasible remediation technologies.

Technology	Water Efficiency	Long-term Effectiveness	Public Safety	Life Cycle Cost	Aesthetic Issues	Customer Acceptance	Waste Management
Pipe Replacement	5	4	5	1	4	2	5
Intermittent Flushing	2	4	4	4	4	4	2
Continuous Flushing	1	4	4	2	3	3	1
Flushing with an Additive	2	2	3	3	3	3	1
POU/POE	5	5	4	1	3	2	4

**Table 7:** Technology priorities under several scenario variants, calculated via Eq. (9). Values in parentheses denote the priority calculated with the closest perfectly consistent criteria comparison matrix.

Technology	Initial case	With cost	With cost, for a School	With cost and waste management
Pipe replacement	**3.75 (3.76)**	3.20 (3.3)	3.48 (3.43)	**3.43 (3.48)**
Intermittent flushing	3.57 (3.55)	**3.51 (3.49)**	3.77 (3.76)	3.42 (3.39)
Continuous flushing	2.69 (2.67)	2.36 (2.40)	2.86 (2.87)	2.33 (2.31)
Flushing with additive	2.70 (2.69)	2.68 (2.66)	2.71 (2.71)	2.60 (2.58)
POU/POE	3.39 (3.40)	3.00 (3.11)	**4.57 (4.57)**	3.16 (3.24)

**Table 8:** Alternate pairwise comparison values for customer acceptance.

Criteria	Customer Acceptance
Water Efficiency	1/3
Long-term Effectiveness	1/7
Public Safety	1/9
Life Cycle Cost	1
Aesthetic Issues	1

**Table 9:** Judgement matrix life cycle cost and customer acceptance scores for a school.

Technology	Life Cycle Cost Score	Customer Acceptance Score
Pipe Replacement	3	2
Intermittent Flushing	5	4
Continuous Flushing	4	3
Flushing with Additive	3	3
POU/POE	5	5
