# Supplementary material for: Informing remediation of benzene contamination in drinking water distribution systems through multi-criteria decision analysis
Source: J Hazard Mater Adv. Author manuscript; Available in PMC 2023 Oct 17. (PMC10581403; doi:10.1016/j.hazadv.2021.100013)
Supplement: Supplementary Material 1 [file NIHMS1924903-supplement-Supplementary_Material_1.docx]

Informing Remediation of Benzene Contamination in Drinking Water Distribution Systems through Multi-Criteria Decision Analysis

Levi M. Haupert^a*^, Jon McDonnell^b^, Kathy Martel^b^, Michael D. Miles^b^, and Matthew L. Magnuson^a^

^a^ U.S. Environmental Protection Agency, Office of Research and Development, Center for Environmental Solutions and Emergency Response, 26 West Martin Luther King Drive, Cincinnati, OH 45268

^b^ CADMUS, 100 Fifth Avenue, Suite 100, Waltham, MA 02451

^*^ Corresponding author: [haupert.levi@epa.gov](mailto:haupert.levi@epa.gov)

https://doi.org/10.1016/j.hazadv.2021.100013

# SupplementaRY Material

Table S1: Criteria-Specific Definitions for Technology Judgement Matrix Scoring

|  | Application-specific Technology Score | | | | |
| --- | --- | --- | --- | --- | --- |
| Criteria | **1** | **2** | **3** | **4** | **5** |
| Aesthetic Issues with Finished Water | This remediation option will definitely cause the finished water to have perceptible tastes, odors, or appearance issues (e.g., red water), and will increase the number of customer complaints. | This remediation option may cause the finished water to have perceptible tastes, odors, or appearance issues (e.g., red water), and may increase the number of customer complaints. | This remediation option may cause minor changes in the water's taste, odor, or appearance but will probably not create any additional customer complaints. | This remediation option may make slight changes in the water's taste, odor, or appearance but will probably not create any additional customer complaints. | This remediation option will not change the aesthetic quality of the finished water. |
| Customer  Acceptance | It will be difficult to achieve customer acceptance for this remediation option. | It may be challenging to achieve customer acceptance but it is expected that acceptance will be achieved with some effort. | Customer acceptance is expected for this option with minimal efforts such as providing information and opportunities for discussion. | Customer acceptance is expected for this option. | It will be easy to get customer acceptance for this remediation option. |
| Energy Efficiency | Using this remediation option will have a strong negative impact on the utility’s ability to meet its efficiency goals. | Using this remediation option will negatively affect the ability of the utility to meet their efficiency goals. | This remediation option is not the most efficient technology but still allows the utility to meet their efficiency goals. | This remediation option includes water or energy efficiency measures. | This remediation option allows the utility to exceed their efficiency goals. |
| Environmental Impact | This remediation option will likely cause moderate to major environmental impacts that will definitely require expensive corrective measures. | The remediation option will likely cause minor environmental impacts that can be addressed through site restoration measures. | This remediation option may cause minor environmental impacts that can be easily addressed through site restoration measures. | This remediation option may cause comparatively insignificant environmental impacts that can be easily addressed through site restoration measures. | This remediation option will not cause any environmental impacts that will need to be addressed. |
| Human Health Impact | This remediation option may require corrective actions to reduce or eliminate potential health risks for customers, operators, or workers through exposure to regulated or unregulated drinking water contaminants (e.g., disinfectants or disinfection byproducts). | This remediation option may require corrective actions to reduce or eliminate potential health risks to system operators and workers that can be mitigated through training and protective equipment. | This remediation option will not impact customer health but may present minor risks to system operators and workers that can be mitigated thru training and protective equipment. | This remediation option will not impact customer health but may present slight risks to system operators and workers that can be mitigated thru training and protective equipment. | This remediation option will not impact the health of customers, operators, or maintenance workers. |
| Impact on Infrastructure | This remediation option is likely to have severe negative impacts on existing piping and other components that will reduce its useful service life. | This remediation option is likely to have moderate impacts (e.g., corrosion) to existing piping and/or other system components. | This remediation option is likely to have moderate impacts (e.g., corrosion) to existing piping and/or other system components. | This remediation option is likely to have minimal impacts (e.g., corrosion) to existing piping or other system components. | This remediation option is likely to have no impacts (e.g., corrosion) to existing piping or other system components. |
| Life Cycle Cost^a^ | This remediation option has very high capital and operations and maintenance costs. | This remediation option has high capital and operations and maintenance costs. | This remediation option has moderate capital and operations and maintenance costs. | This remediation option has low capital and operations and maintenance costs. | This remediation option has low capital and operations and maintenance costs. |
| Long-term Effectiveness | This remediation option will have to be performed multiple times to completely remove the contaminant to desired levels. | This remediation option may have to be repeated at least a second time to completely remove the contaminant to desired levels. | This remediation option may remove the contaminant to levels close to the desired levels. | This remediation option will likely remove the contaminant to levels below the desired levels. | This remediation option will, in most circumstances, completely remove the contaminant to levels below the desired levels. |
| Operator or Worker Safety | This remediation option presents significant safety hazards to utility operators and workers and will require significant additional training, signage, and PPE and may preclude operators and workers from doing their jobs. | This remediation option presents moderate safety hazards to operators and workers and will require additional training, signage, and PPE but probably does not preclude operators and workers from doing their jobs. | This remediation option includes some safety hazards for operators/workers but can be managed through training, signage, and personal protective equipment (PPE). | This remediation option presents only minor safety hazards that can be addressed through training, signage, and PPE. | This remediation option does not present any additional safety hazards to operators or workers beyond normal training, signage, and PPE. |
| Public Safety | This remediation option presents significant safety hazards to the public. | This remediation option presents moderate safety hazards to the public. | This remediation option includes some safety hazards to the public. | This remediation option presents only minor safety hazards to the public. | This remediation option does not present any additional safety hazards to the public. |
| Regulatory Impacts and Considerations | This remediation option may cause violations of Safe Drinking Water Act and other regulations. | This remediation option may cause violations of Safe Drinking Water Act or other regulations. | This remediation option may have moderate regulatory impacts. | This remediation option may have minor regulatory impacts. | This remediation option will likely not cause simultaneous compliance issues with other regulations. |
| Timeframe^b^ | This remediation option will take a significant amount of time to execute (greater than 6 months). | This remediation option can be executed in the next 4-6 months. | This remediation option can be executed in the next 1-3 months. | This remediation option can be executed within the next 1 to 4 weeks. | This remediation option can be executed within the next week. |
| Waste Management Considerations | This remediation option produces a solid waste stream requiring special disposal and an aqueous waste stream that can’t be discharged to the sewer system after pre-treatment, so additional treatment, other off-site disposal methods, and/or permits may be required. | This remediation option will produce a solid waste stream requiring special disposal and an aqueous waste stream that may require treatment before it can be discharged to the sewer system. A permit may be required for waste disposal. | This remediation option may produce a solid waste stream with no special disposal requirements or an aqueous waste stream that can be discharged to the sewer system without a permit. | This remediation option may produce a solid waste stream or an aqueous waste stream, but it can be disposed onsite. | This remediation option will not produce a waste stream that requires management. |
| Water Efficiency | Using this remediation option will negatively affect the utility’s ability to meet its efficiency goals. | Using this remediation option may negatively affect the utility’s ability to meet its efficiency goals. | This remediation option is not the most efficient technology but still allows the utility to meet its efficiency goals. | This remediation option includes water or energy efficiency measures. | This remediation option allows the utility to exceed its efficiency goals. |
| Custom Criteria | [Determined by user] | [Determined by user] | [Determined by user] | [Determined by user] | [Determined by user] |

a. Life cycle costs do not include cost associated with the water system being out of service, e.g., costs included with supplying alternative water supplies or lost productivity in the community due to lack of available water.

b. Specific timetables listed are examples. Users may substitute them with more appropriate timetables to best suit their needs.
